# Applications of nanomaterials in endometriosis treatment

**DOI:** 10.3389/fbioe.2023.1184155

**Published:** 2023-05-09

**Authors:** Jiang Yuxue, Sun Ran, Fan Minghui, Sheng Minjia

**Affiliations:** The Reproductive Medical Center, China-Japan Union Hospital of Jilin University, Changchun, Jilin, China

**Keywords:** endometriosis, nanotechnology, nanomaterials, delivery system, gene therapy

## Abstract

Endometriosis is a common disease of the reproductive system in women of childbearing age with an unclear pathogenesis. Endometriosis mainly manifests as dysmenorrhea, abdominal pain, and infertility. Currently, medical therapy and surgical treatment are usually used for endometriosis treatment. However, due to the high recurrence rate and many complications, it has greatly affected patients’ quality of life. Nanotechnology is a new technology that mainly investigates the characteristics and applications of nanomaterials. To date, nanotechnology has received widespread attention in the field of biomedicine. Nanomaterials can not only be used as drugs to treat endometriosis directly, but also enhance the therapeutic effect of endometriosis by delivering drugs, siRNA, antibodies, vesicles, etc. This review comprehensively discusses nanomaterial-based therapies for endometriosis treatment, such as nanomaterial-based gene therapy, photothermal therapy, immunotherapy, and magnetic hyperthermia, which provides a theoretical reference for the clinical application of nanotechnology in the treatment of endometriosis.

## 1 Introduction

Endometriosis is a common, chronic, inflammatory gynaecological disease characterized by endometriotic lesions present outside the uterine cavity. The main clinical symptoms of endometriosis are dysmenorrhea, pelvic pain, and infertility (Horne and Missmer, 2022). The incidence rate of endometriosis in women of reproductive age is approximately 10% (Zondervan et al., 2020). Although endometriosis is a benign disease, ectopic cells often exhibit infiltrating growth, similar to malignant tumours. According to the different site of occurrence, it can be divided into ovarian, peritoneal and deep infiltrating endometriosis (Nisolle and Donnez, 2019). The pathogenesis of endometriosis is extremely complex, including oxidative stress and angiogenesis (Saunders and Horne, 2021; Clower et al., 2022). Early diagnosis of endometriosis is difficult and challenging, and currently relies on ultrasound, magnetic resonance imaging and hysteroscopy. Hysteroscopy is widely considered the gold standard for the diagnosis of endometriosis. At present, the treatment of endometriosis involves both medical therapy and surgical treatment. Medical therapy generally reduces estrogen levels to alleviate pain and other symptoms. But due to the long-term use of hormone suppressive drugs, patients may experience side effects such as nausea, headaches, vasomotor symptoms, vascular dryness, sleep and disturbance (Saunders and Horne, 2021). Surgical treatment can effectively remove the lesions. However, the 5-year postoperative recurrence rate reaches 40%–50% (Guo et al., 2009), seriously affecting patients’ quality of life.

Nanotechnology refers to the exploration and utilization of materials on the nanometre scale in various fields (Koopmans and Aggeli, 2010). Nanomaterials, which are approximately 1–100 nm in size, have many advantages, such as the targeting ability, good biocompatibility, stability, and extremely low toxicity (Riehemann et al., 2009). Many studies have proven that nanomaterials, as therapeutic agents or drug delivery carriers, have great applications in the medical field, to treat conditions such as inflammatory diseases, infectious diseases, cardiovascular diseases, and cancer (Kim et al., 2010; Psarros et al., 2012; Liu et al., 2018; Liu et al., 2019; Liu et al., 2020; Liu et al., 2021a; Liu et al., 2021b; Moses et al., 2021; Wang et al., 2022; Zhang et al., 2022; Xu et al, 2023a; You et al., 2023; Russell et al., 2023). To date, nanotechnology-based approaches have been valid in non-invasive diagnosis and treatment of endometriosis, which effectively target ectopic tissues and cells without causing systemic effects. Therefore, Nanotechnology has potential applications for the treatment of endometriosis.

**FIGURE 1:**
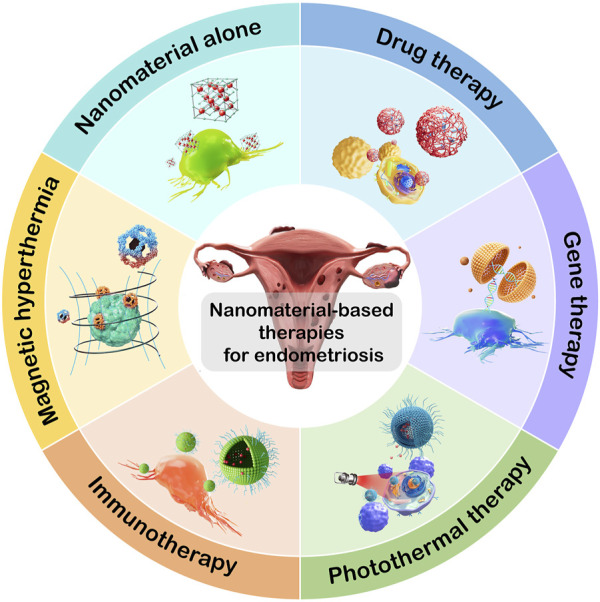
Nanomaterial-based therapies for endometriosis treatment.

In this review, we focus on the impact of nanomaterial-based strategies in the treatment of endometriosis in recent years, including nanomaterials alone, nanomaterial-based drug therapy, gene therapy, photothermal therapy, immunotherapy, and magnetic hyperthermia (Figure 1; Table 1).

**TABLE 1:** Summary of the applications of nanomaterials in endometriosis treatment.

Application	Nanomaterial (with or without modification)	Cargo molecule	Animal model	Results	Author, year
**Therapeutic agent**					
	Nanoceria	-	Mouse (i.p. injection of uterine tissue)	reduced endometrial lesions via decreasing oxidative stress markers and angiogenesis	Chaudhury et al. (2013)
**Drug therapy**					
	PLGA	Epigallocatechin gallate and doxycycline	Mouse (i.p. injection of uterine tissue)	Mitigation endometriosis by lowering the level of oxidative stress, angiogenesis, and MMP activity	Singh et al. (2015)
	CSOSA/NLC	A-317491	Rat (sutured uterine tissue onto the mesenteric arteries and peritoneum)	Alleviated mechanical and thermal hyperalgesia, with increases in the MPT and HSL	Yuan et al. (2017)
	PLGA	Copaiba oleoresin	-	-	de Almeida Borges et al. (2018)
	PCL-PEG	Curcumin	Mouse (i.p. injection of uterine tissue)	Decreased endometrial glands, stroma, and infiltrating inflammatory cell	Boroumand et al. (2019)
	Silver nanoparticles	PL1-MMAE	-	-	Simón-Gracia et al. (2021)
**Gene therapy**					
	CSO-SA	PEDF	Rat (sutured uterine tissue onto the peritoneum)		Zhao et al. (2012)
	(CSO-PEI) HA	AQP2-siRNA	Rat (sutured uterine tissue onto the peritoneum)	Reduced the endometriotic lesion sizes with low expression of CD44	Zhao et al. (2016)
	(PEI–SA) HA	Beclin-1	Mouse (i.p. injection of uterine tissue)	repressed the growth of endometriotic cysts by enhancing autophagy with Beclin-1 expression	Zhao et al. (2022)
	PAMAM	Endostatin	Mouse (s.c. injection of endometrial cell)	Inhibited the growth of endometriotic lesions, reduced CD31 expression	Wang et al. (2014)
	PEI–PEG–RGD	MiR-200c	Rat (i.p. injection of uterine tissue)	Reduced ectopic endometrial cyst volume	Liang et al. (2017)
	CPP (cell-penetrating peptides)	RRM2	-	-	Kiisholts et al. (2021)
	Exosome	MiR-214; miR-213-3p	Mouse (i.p. injection of human eutopic endometrial tissue); Mouse (i.p. injection of uterine tissue)	Inhibited fibrosis by targeting CCN2	Wu et al., 2018; Zhang et al., 2021
**Photothermal therapy**					
	PEG-PCL	SiNc	Mouse (s.c. injection of monkey endometriotic tissue)	Eliminated the endometriotic lesions	Moses et al. (2020)
	HAuNS (hollow gold nanospheres)	TNYL	Mouse (s.c. injection of rat uterine tissue)	Inhibited the growth of the lesions, destroyed the structure of the lesions, decreased levels of TNF-α and estradiol	Guo et al. (2017)
**Immunotherapy**					
	PLGA	Anti-CTLA-4	Mouse (sutured Endometrium tissue to the peritoneal wall)	Decreased the percentage of CD4^+^CD25^+^ Treg cells, restricted ectopic endometrial cell proliferation and invasion by repressing IL-10 and TGF-beta secretion	Liu et al. (2017)
	M1NVs (nanovesicles derived from M1 macrophages)	-	Mouse (i.p. injection of uterine tissue)	Inhibited endometriosis by repolarizing M2 macrophages to M1 macrophages	Li et al. (2021)
**Magnetic hyperthermia**					
	Hexagonal iron oxide nanoparticles coated by PEG-PCL	KDR	Mouse (s.c. injection of macaque endometrium tissue)	KDR-targeted MN accumulated in endometriotic grafts, increased the temperature under an AMF, and eliminated endometriotic lesions	Park et al. (2022)

## 2 Nanomaterials alone for endometriosis treatment

Nanomaterials are often used as drug delivery carriers, but due to the characteristics of some nanomaterial, it can also be used as a therapeutic agent to treat endometriosis (Chaudhury et al., 2013). It was found that cerium oxide nanoparticles (nanoceria) play a pivotal role in the treatment of diseases related to oxidative stress (Celardo et al., 2011). Therefore, Chaudhury et al. investigated whether nanoceria could treat endometriosis. Compared with control mice, nanoceria significantly reduced endometrial lesions via decreasing oxidative stress markers and angiogenesis in endometriosis-induced mice, demonstrating that nanoceria may have potential as a treatment for endometriosis (Chaudhury et al., 2013).

## 3 Nanomaterial-based drug therapy

Although available drugs have good efficacy in the treatment of endometriosis, there are still some limitations, such as poor stability, low biological activity and weak targeting. Therefore, nanomaterials, as drug delivery carriers, have the ability to compensate for the shortcomings of these drugs for endometriosis treatment.

Both epigallocatechin gallate (EGCG) and doxycycline (Dox) have been reported to have antioxidant and antiangiogenic properties and inhibitory effects on matrix metalloproteinases (MMPs) (Acharya et al., 2004; Sapadin and Fleischmajer, 2006; Singh et al., 2011), and have thus been applied in the treatment of endometriosis (Akkaya et al., 2009; Ricci et al., 2013). However, the instability of EGCG and Dox has limited their therapeutic application (Mochizuki et al., 2002; Misra et al., 2009). Because nanomaterials can improve the stability and bioavailability of medicines, Poly (lactic-co-glycolic acid) (PLGA) is an appropriate candidate for drug delivery for the treatment of various diseases (Makadia and Siegel, 2011). Therefore, Singh et al. synthesized single drug-loaded PLGA nanoparticles (EGCG NPs, DOX NPs) and dual drug-loaded nanoparticles (DOX-EGCG NPs) to treat mice with endometriosis (Singh et al., 2015). The DOX-EGCG NPs were more effective than EGCG NPs or DOX NPs in the treatment of endometriosis, lowering the level of oxidative stress, angiogenesis, and MMP activity in mice with induced endometriosis (Singh et al., 2015).

A-317491 is a P2X3 receptor antagonist that can relieve inflammation and neuropathic pain. However, due to its short half-life and poor biodistribution, its analgesic effect in endometriosis has been seriously hindered (Hansen et al., 2012). Yuan et al. synthesized CSOSA/NLC/A-317491, a chitosan oligosaccharide-stearic acid (CSOSA) polymer micelle-coated nanostructured lipid carrier (NLC) for A-317491 drug delivery (CSOSA/NLC/A-317491) (Yuan et al., 2017). In endometriotic rats, CSOSA/NLC/A-317491 remarkably alleviated mechanical and thermal hyperalgesia for a long period of time, with increases in the mechanical pain threshold (MPT) and heat source latency (HSL) (Yuan et al., 2017). Therefore, CSOSA/NLC/A-317491 may be an effective treatment strategy for endometriosis pain.

There are also some small molecules that are used to treat endometriosis, such as copaiba oleoresin (CPO) (de Almeida Borges et al., 2018). CPO, a natural product of trees of the genus Copaifera, has the ability to inhibit the proliferation of human endometrial stromal cells (Henriques da Silva et al., 2015). The authors investigated whether PLGA nanoparticles containing CPO could further decrease the viability of human endometrial stromal cells (de Almeida Borges et al., 2018). They found that PLGA nanoparticles containing CPO reduced the cell viability in the ectopic endometrium and eutopic endometriotic lesions of patients with endometriosis.

Many studies have been published on the wide effects of curcumin on various diseases, such as inflammatory bowel disease, arthritis, all kinds of cancer, and endometriosis (Jurenka et al., 2009; Gupta et al., 2013). However, because of the low bioavailability of curcumin (Arablou and Kolahdouz-Mohammadi, 2018), Boroumand et al. synthesized curcumin-loaded poly *ε*-caprolactone (PCL) and polyethylene glycol (PEG) nanofibers to investigate whether they could increase the release of curcumin *in vitro* and *in vivo* (Boroumand et al., 2019). The results showed that almost 50% of the curcumin was released from curcumin-loaded PCL-PEG nanofibers over 30 days *in vitro*, and the curcumin-loaded nanofibers significantly ameliorated the endometriosis with related histological characteristics, such as the reduction of endometrial glands, stroma, and infiltrating inflammatory cells.

**FIGURE 2:**
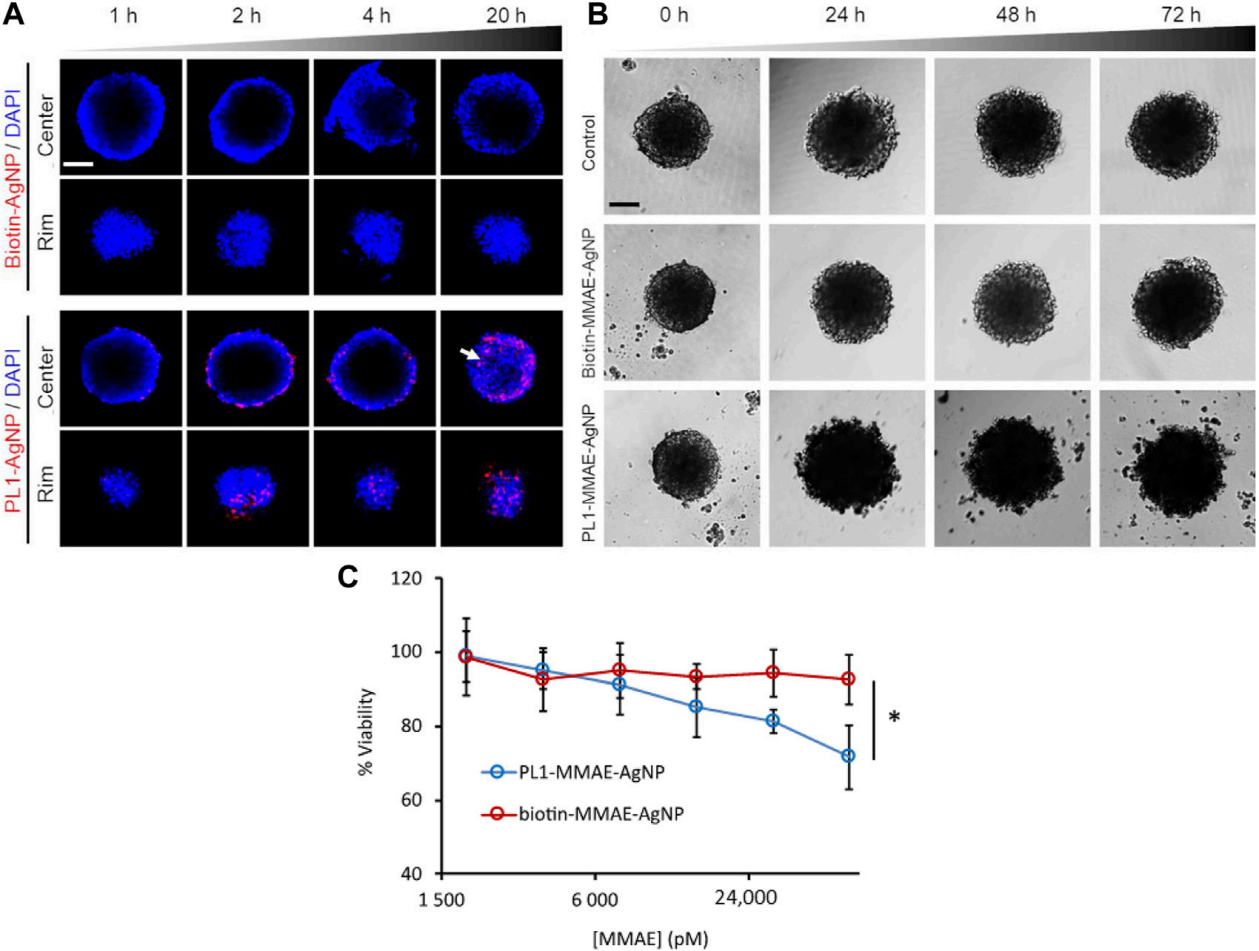
PL1-AgNPs internalized into 12Z spheroids, and PL1-MMAE AgNPs enhanced the cytotoxicity to 12Z spheroids. **(A)** Confocal fluorescence images of cellular internalization of PL1-AgNPs or biotin-AgNPs in12Z spheroids for 1, 2, 4, and 20 h. **(B)** Morphological images of 12Z spheroids incubated with PL1-MMAE-NPs or biotin MMAE-AgNPs for 24, 48, and 72 h. **(C)** Cell viability of 12Z spheroids incubated with different concentrations of PL1-MMAE-AgNPs or biotin-MMAE-AgNPs for 72 h *p < 0.05. Reprinted with permission from (Simón-Gracia et al., 2021).

Consistent with solid tumours, the expression of some angiogenic extracellular matrix proteins in endometriosis was abnormal, such the high expressed tenascin C domain C (TNC-C) and fibronectin extra domain-B (Fn-EDB). The authors showed in a previous study that a peptide that binds to TNC-C and Fn-EDB (PL1 peptide) effectively inhibited the growth of glioblastoma (Lingasamy et al., 2019). Considering the similarities in the pathogeneses of tumours and endometriosis, the author inferred the strong effect of the PL1 peptide to target endometriosis for its treatment in this study (Simón-Gracia et al., 2021). First, they synthesized the PL1 peptide with silver nanoparticles (PL1-AgNPs) and found that PL1-AgNPs were internalized into 12Z and HESC cells, which highly expressed the PL1 peptide receptors TNC-C and Fn-EDB. Then, to test the targeted killing effect of the PL1-AgNPs, a potent antimitotic agent, monomethyl auristatin E (MMAE), was integrated into the PL1-AgNPs (PL1-MMAE-AgNPs). The results showed that PL1-MMAE-AgNPs significantly damaged morphology and suppressed the survival in 12Z spheroids (Figure 2). Moreover, PL1-AgNPs bound to human peritoneal endometriotic lesions where both TNC-C and Fn-EDB were expressed. As described above, PL1-AgNPs have the potential to become a tool for the targeted treatment of endometriosis.

## 4 Nanomaterial-based gene therapy

Gene therapy is an approach to disease treatment that transfers specific genes to target cells and modifies or compensates for gene defects. In recent years, gene therapy has been widely used in the treatment of various conditions, including inflammation, nervous system diseases, and cancer. The key to gene therapy is a safe and effective carrier system, therefore, nanocarriers have attracted the attention of researchers because of their characteristics of high targeting specificity and safety (Yin et al., 2014). To date, many studies have shown that gene therapy based on nanotechnology can be applied in the treatment of endometriosis. Nanocarriers involved in endometriosis gene therapy include polymeric nanoparticles, cell-penetrating peptides (CPPs), and extracellular vehicles (EVs).

### 4.1 Polymeric nanoparticles as nanocarriers for endometriosis gene therapy

Polymeric nanoparticles are produced by natural polymers or synthetic polymers, such as chitosan oligosaccharide (CSO), polyetherimide (PEI), polyamidoamine (PAMAM), and poly (lactic-co-glycolic acid) (PLGA). Polymeric nanoparticles possess many properties suitable for the nano delivery of nucleic acids due to their easy surface modification, good stability, high safety and favourable biocompatibility (Mendes et al., 2022). Herein, single or mixtures of multiple polymers were used as carriers for endometriosis gene therapy.

Chitosan is widely used for plasmid delivery due to its biocompatible, low immunogenic, and biodegradable nature, but its transfection efficiency is very low (Dass et al., 2007). Stearic acid is an endogenous long-chain saturated fatty acid that forms a polymer micelle that is characterized by its great membrane permeability, rapid intracellular uptake and site-specific delivery in aqueous media with polysaccharides (Foged et al., 2007). Thus, Zhao et al. (2012) synthesized and used CSO-SA to deliver PEDF (pigment epithelium-derived factor) to inhibit angiogenesis in endometriotic lesions. The CSO-SA/PEDF nanoparticles showed no toxicity to the reproductive organs, and the sizes of the endometriotic lesions and atrophy and degeneration of the ectopic endometrium decreased significantly (Zhao et al., 2012). Moreover, microvessel density decreased, and apoptosis increased (Zhao et al., 2012). Therefore, a glycolipid-like structure micelle-mediated PEDF gene delivery system could be used for the treatment of endometriosis.

Based on a previous study (Zhao et al., 2012), Zhao et al. synthesized another novel polymeric nanoparticle gene delivery system for endometriosis treatment (Zhao et al., 2016). They synthesized a gene carrier with CSO-PEI, hyaluronic acid (HA), and small interfering RNA (siRNA). HA can bind to CD44, which is overexpressed in endometriotic lesions. AQP2 is related to the progression of endometriosis, so AQP2-siRNA was applied in this study. The results showed that (CSO-PEI/siRNA) HA nanoparticles significantly inhibited the development of endometriosis with low expression of CD44 in a rat model. Thus, it was confirmed that (CSO-PEI/siRNA) HA is a potential tool for endometriosis treatment.

Autophagy has an essential role in many diseases, including various cancers and endometriosis (Yang et al., 2019; Ogasawara et al., 2020). Beclin-1, a key regulator of autophagy, has been reported to have low expression in endometrial hyperplasia and endometrioid cancer (Zhao et al., 2006). Therefore, Zhao et al. also investigated the role of autophagy in the treatment of endometriosis with nanoplatforms. In a previous study, Zhao et al. prepared PEI-SA nanoplatforms to treat ovarian cancer (Zhao et al., 2019). The authors modified the nanoplatforms with nucleotides and enclosed them with HA to investigate the therapeutic effect on endometriosis (Zhao et al., 2022). As a result, the (PEI–SA/DNA) HA gene delivery system repressed the growth of endometriotic cysts by enhancing autophagy with Beclin-1 expression. Therefore, (PEI–SA/DNA) HA gene carriers are a new and promising way to cure endometriosis.

As angiogenesis is a main cause of the development of endometriosis, it is necessary to effectively inhibit angiogenesis when treating this condition. Endostatin is a well-characterized inhibitor of angiogenesis, but the use of endostatin is limited because of its short-lived effects (Becker et al., 2006). Polyamidoamine (PAMAM) dendrimers are recently developed gene vectors that are commercially available for use in gene transfer (Shakhbazau et al., 2010). To improve endometriosis treatment, Wang et al. used an endostatin-loaded PAMAM (PAMAM-Es) plasmid as a gene vector in a non-invasive animal model (Wang et al., 2014). Compared to Lipofectamine Es, PAMAM-Es significantly inhibited the growth of endometriotic lesions. The angiogenesis biomarkers CD31 and VEGF were also assessed to detect the antiangiogenic efficiency of PAMAM-Es. The expression of CD31 was found to be reduced, which demonstrated that PAMAM-Es can treat endometriosis through an antiangiogenic mechanism.

In another study, PEI was used to form conjugates with PEG and arginine–glycine–aspartic acid (RGD) peptides (generating PEI–PEG–RGD conjugates) by reaction of the cysteine group with maleimide to deliver miR-200c in a rat model of endometriosis (Liang et al., 2017). An miR-200c mimic and inhibitor were delivered into endometriotic lesions by the PEI–PEG–RGD conjugates, as confirmed by near-infrared imaging (Liang et al., 2017). The ectopic endometrial cyst volume was significantly reduced after the miR-200c mimic was delivered by this conjugate but increased after treatment with the miR-200c inhibitor (Liang et al., 2017).

### 4.2 CPPs as nanocarriers for endometriosis gene therapy

**FIGURE 3:**
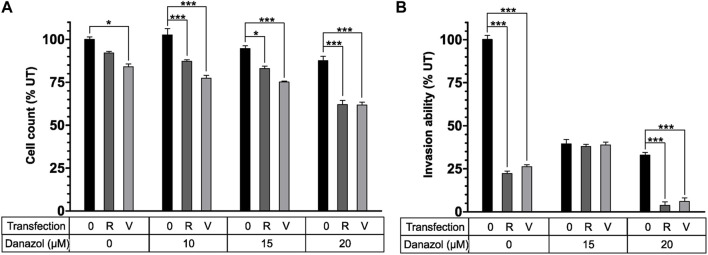
The combination of NF70/siRNA NP and danazol inhibited endometriotic cell proliferation and invasion. **(A)** The number of peritoneal endometriotic cells treated with NF70/siRNA NP and danazol. **(B)** Invasion of peritoneal endometriotic cells treated with NF70/siRNA NP and danazol by the extracellular matrix. *p < 0.05, ***p < 0.001.0—no NP transfection; R—NF70/siRRM2; V—NF70/siVEGF. Reprinted with permission from (Kiisholts et al., 2021).

CPPs are small molecular peptides that can penetrate cell membranes and deliver proteins and nucleic acids into cells (Guidotti et al., 2017). Among them, the nanocarriers PepFect6 (PF6) and NickFect70 (NF70) are widely used in siRNA delivery (Andaloussi et al., 2011; Porosk et al., 2019). Kiisholts et al. studied the effect of both CPPs with siRNA nanoparticles in the treatment of endometriosis and identified that RRM2 might be a new potential target for endometriosis treatment (Kiisholts et al., 2021). In peritoneal and ovarian endometrial cells, CPP/siRNA nanoparticles reduced the mRNA and protein levels of RRM2 and VEGF and effectively inhibited cell division, leading to cell cycle arrest in G1/S phase. In a three-dimensional culture model, CPP/siRNA nanoparticles inhibited the invasion and migration of endometriotic cells. In addition, the combination of CPP/siRNA nanoparticles and the endometriosis drug danazol significantly inhibited the proliferation and invasion of endometriotic cells (Figure 3). However, further research is needed to confirm the role of CPP/siRNA nanoparticles, especially in endometriosis animal models.

### 4.3 EVs as nanocarriers for endometriosis gene therapy

EVs are natural nanoparticles secreted by a variety of cells that have a double-layer lipid membrane structure. EVs carry many biologically active molecules, such as proteins, lipids, DNA, or mRNA, and participate in the regulation of cell function. EVs mainly include exosomes, microvesicles, and apoptotic bodies. EVs have many advantages, including good biocompatibility, low immunogenicity, and low toxicity. Therefore, as shining stars in nanomedicine, EVs are considered to be promising nanocarriers for drug delivery systems (Wu et al., 2021; Fang et al., 2022). In recent years, it has been reported that many miRNAs and lncRNAs carried by EVs participate in endometriosis gene therapy (Wu et al., 2018).

The excessive deposition of extracellular matrix in endometrial glands leads to endometrial fibrosis, which affects the function of the endometrium. Thus, it is very important to explore endometriosis fibrosis treatment. The results from two different research groups revealed that exosomal miR-214 or miR-214-3p derived from ectopic endometriosis stromal cells inhibited fibrosis by targeting CCN2, which is closely related to fibrogenesis (Wu et al., 2018; Zhang et al., 2021). Moreover, Zhang and his colleagues further reported that exosomes played a crucial role in the transmission of miR-214-3p for fibrosis treatment (Zhang et al., 2021).

Furthermore, many studies have proven that some miRNAs, lncRNAs, or proteins transmitted by exosomes may be potential targets for the treatment of EM, such as miR-22-3p, lncRNA CHL1-AS1, lncRNA aHIF and VEGF-C (Qiu et al., 2019; Li et al., 2020; Zhang et al., 2020; Liu et al., 2021c).

Therefore, there is great potential for using EVs as delivery vectors for the treatment of endometriosis.

## 5 Nanomaterial-based photothermal therapy

In recent years, photothermal therapy has become a new and promising modality for tumour treatment. Photothermal therapy involves the delivery of the photothermal agent to the tumour site and irradiation with a near-infrared (NIR) laser to raise the temperature of the tumour tissue above 42 °C for completely eradication of the tumour cells (Anbil et al., 2013; Duong et al., 2017). The method requires a short period of time and has few toxic side effects. A variety of nanomaterials have been developed for photothermal therapy of tumours (Xu et al., 2023b; Schneider et al., 2023; Tan et al., 2023). Because the pathogenesis of endometriosis is similar to that of tumours, the use of photothermal therapy based on nanomaterials has also been reported to treat endometriosis (Guo et al., 2017; Moses et al., 2020).

Moses et al. (2020) prepared nanoplatforms with silicon phthalocyanine (SiNc) coated by PEG-PCL-based polymeric nanoparticles (SiNc-NPs) that were utilized for real-time NIR fluorescence imaging and photothermal therapy. They evaluated the photothermal therapy efficacy of SiNc-NPs in endometriosis treatment *in vitro* and *in vivo*. The results showed that SiNc-NPs killed more than 95% of the endometriotic macaque endometriotic stromal cells after incubation with SiNc-NPs for 2 days followed by exposure to 780 nm NIR light for 15 min. *In vivo*, SiNc-NPs were injected intravenously into the endometriosis model mice for 1 day, and then NIR light was used to illuminate the endometriotic grafts for 15 min. As a result, SiNc-NPs completely eliminated the endometriotic lesions without any side effects. Therefore, these results revealed that SiNc-NPs may be a safe and effective nanoplatform for endometriosis treatment.

Guo et al. assessed the photothermal therapy efficacy of hollow gold nanospheres (HAuNSs) for endometriosis (Guo et al., 2017). HAuNS, as a potential photosensitive agent, is widely utilized for photothermal therapy (Wang et al., 2015). TNYL specifically recognizes the EphB4 receptor, which is highly expressed in endometriotic lesions. Therefore, the authors developed HAuNS and TNYL-conjugated HAuNS (TNYL-HAuNS) to treat endometriosis (Guo et al., 2017). These nanoparticles were injected via the tail vein into the endometriosis model mice. The results showed that TNYL-HAuNS accumulated more in the endometriotic lesions than HAuNS alone, owing to the high-affinity binding between the TNYL peptide and EphB4 receptor. After NIR laser irradiation, TNYL-HAuNS strongly inhibited the growth of the lesions and more severely destroyed their structure. Furthermore, the levels of TNF-α and estradiol also significantly decreased in mice treated with TNYL-HAuNS and NIR laser irradiation. Thus, the use of TNYL-HAuNS may be a new approach for photothermal therapy of endometriosis.

## 6 Nanomaterial-based immunotherapy

Immunotherapy plays a critical role in the treatment of various tumours. Tumour immunotherapy inhibits the growth of tumour cells by improving innate and adaptive immune responses. Due to the nontoxicity, high biosafety, and excellent targeted drug delivery of nanomaterials, nanoimmunotherapy has received widespread attention in tumour immunotherapy (Li et al., 2022). Since the pathogenesis of endometriosis is similar to that of a solid tumour, many immune factors are closely related to the occurrence and development of endometriosis. Therefore, it can be inferred that immunotherapy may be applied to treat endometriosis. Currently, it has been reported that nanoimmunotherapy has a profound positive effect on the treatment of endometriosis (Liu et al., 2017; Li et al., 2021).

Numerous studies have shown that the development of endometriosis is related to immunologic factors and involves a significant increase in the number of CD4^+^ CD25^+^ regulatory T cells (Podgaec et al., 2012; Greaves et al., 2014). Anti-CTLA-4 (cytotoxic T lymphocyte antigen-4) has been used as an immune checkpoint inhibitor for inhibiting CD4^+^ CD25^+^ regulatory T-cell activation (Zou and Chen, 2008). As PLGA-based drug delivery systems have a variety of applications for different diseases, Liu et al. synthesized PLGA/anti-CTLA-4 nanoparticles to determine whether they play an important role in the treatment of endometriosis (Liu et al., 2017). The results showed that PLGA/anti-CTLA-4 significantly decreased the percentage of CD4^+^CD25^+^ Treg cells in peritoneal fluid in a mouse model of endometriosis. Furthermore, PLGA/anti-CTLA-4 restricted ectopic endometrial cell proliferation and invasion by repressing IL-10 and TGF-beta secretion by CD4^+^CD25^+^ Treg cells.

Li et al. (2021) demonstrated that nanovesicles (NVs) derived from M1 macrophages (M1NVs) suppressed the development of endometriosis. NVs, as natural nanoparticles, are generated by the serial extrusion of cells and have been proven to have significant regulatory effects in many diseases (Choo et al., 2018; Dad et al., 2021). In this study, the authors prepared M1NVs to evaluate their effects on endometriosis *in vitro* and *in vivo* (Li et al., 2021). The results indicated that M1NV treatment significantly suppressed the migration and invasion of ectopic endometrial stromal cells from endometriosis patients. Furthermore, M1NV treatment inhibited endometriosis by repolarizing M2 macrophages to M1 macrophages without any side effects. Thus, M1NVs, as immunological factors, may be a potential tool for endometriosis treatment. Regrettably, although M1NVs are also potential nanocarriers, there have been no further investigations into which molecule carried in the M1NVs had an effect on endometriosis treatment in this study.

## 7 Nanomaterial-based magnetic hyperthermia

Magnetic nanoparticle hyperthermia is a novel, non-invasive method for tumour treatment. Magnetic nanoparticles are delivered to the tumour region and induce the generation of heat under an alternating magnetic field (AMF); then, the tumour cells are killed when the local temperature exceeds 42°C (Kumar and Mohammad, 2011; Hilger, 2013). Although magnetic nanoparticle hyperthermia is widely used in the treatment of tumours, there is little related research on their use in endometriosis treatment.

**FIGURE 4:**
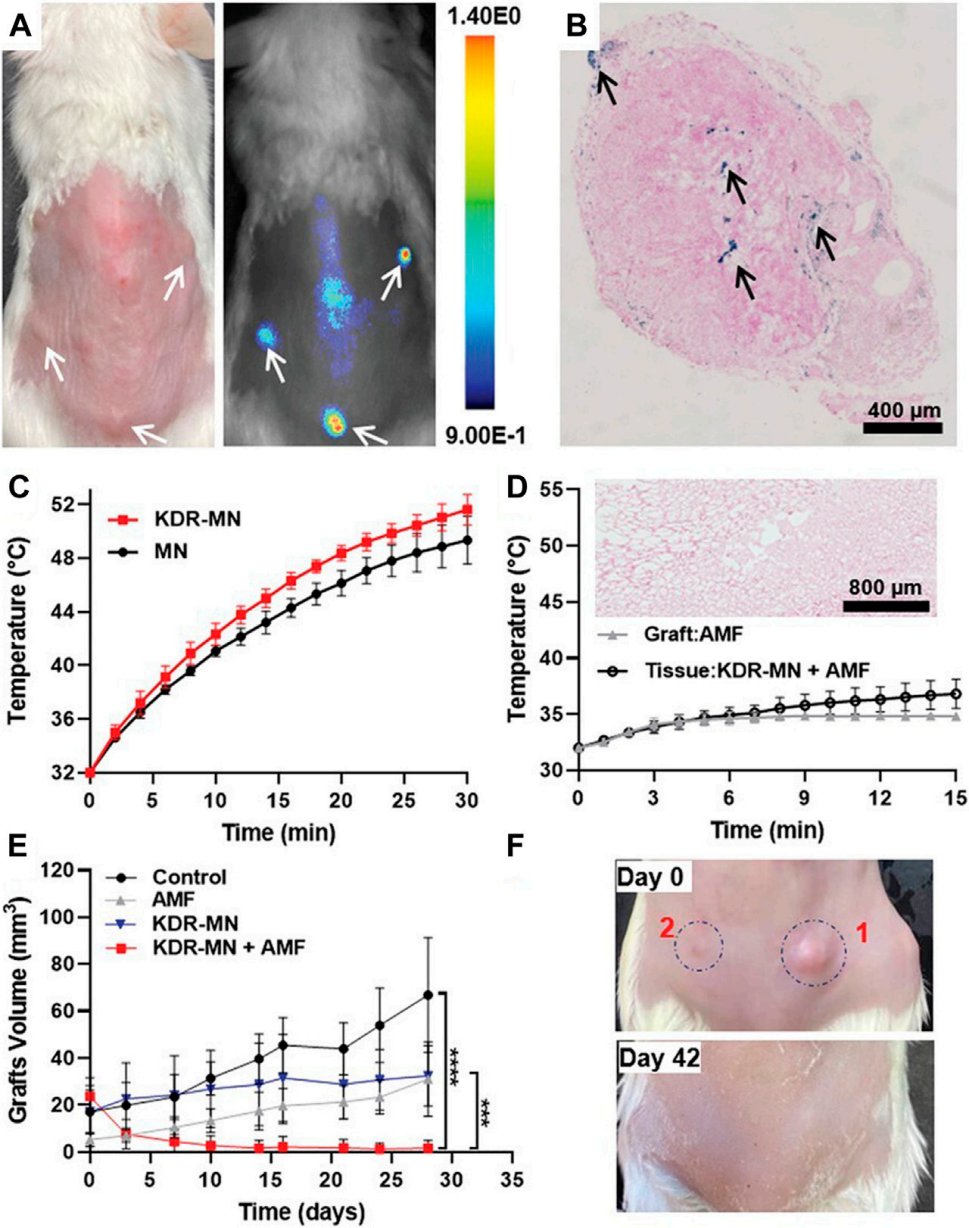
KDR-MN treatment eliminated endometriotic grafts. **(A)** The photograph (left) and NIR fluorescence image (right) of the mouse model of endometriosis treated with NIR fluorescence dye loaded KDR-MN. **(B)** Prussian blue-stained sections of endometriosis grafts from the mouse model of endometriosis treated with KDR-MN. **(C)** Temperature distribution of endometriotic grafts treated with non-targeted MN and KDR-MN under AMF. **(D)** Temperature distribution of endometriotic grafts and tissues adjacent to grafts treated with KDR-MN under AMF. **(E)** The growth of endometriotic grafts treated with KDR-MN with AMF exposure or not. **(F)** Photographs of endometriotic grafts in the mouse model of endometriosis treated with single hyperthermia. ***p < 0.001, ****p < 0.0001. Reprinted with permission from (Park et al., 2022).

Encouragingly, Park et al. recently demonstrated the efficiency of magnetic nanoparticle hyperthermia in endometriosis for the first time (Park et al., 2022). They first developed hexagonal iron oxide nanoparticles coated by poly (ethylene glycol)-block-poly (ε-caprolactone) (PEG-PCL)-based nanocarriers, which were then modified with peptides to target vascular endothelial growth factor receptor 2 (VEGFR-2, also known as KDR). The developed nanoparticles had the advantages of high heating efficiency and targeting specificity to endometriotic cells. The authors next evaluated the therapeutic efficiency of KDR-targeted magnetic nanoparticles (MN) *in vitro* and *in vivo*. Compared with nontargeted MN, KDR-targeted MN raised the temperature above 46 °C more quickly and the effect lasted for a longer time, so the nanoparticles killed more macaque endometriotic cells in the presence of an AMF. In the *in vivo* experiment, a clinically relevant dose of KDR-targeted MN (3 mg per kg) was intravenously injected into endometriosis model mice. The results revealed that the KDR-targeted MN accumulated in endometriotic grafts, increased the temperature under an AMF, and eventually eliminated endometriotic lesions (Figure 4).

## 8 Discussion

Endometriosis has seriously affected the quality of life of women of childbearing age. Therefore, it is essential to develop effective approaches to treat this disease. In recent years, an increasing number of researchers have exploited nanotechnology to improve the therapeutic effect of endometriosis treatment. Because nanomaterials have good biocompatibility, high targeting abilities, easy modification, low toxicity and other advantages, they can be used as delivery carriers to participate in the targeted delivery of drugs, thus improving the efficacy of endometriosis treatment. A large amount of evidence has shown that nanotechnology plays an important role in the treatment of endometriosis with traditional therapy, gene therapy, immunotherapy, photothermal therapy and magnetic hyperthermia.

Currently, the endometriosis animal models used in most studies are mouse or rat models. As the pathogenesis of endometriosis is complex, these models are not sufficient to clarify the role of nanotechnology in spontaneous endometriosis. Therefore, it is urgent to develop a spontaneous animal model similar to human endometriosis, especially a nonhuman primate animal model.

Moreover, in the treatment of endometriosis, the biological safety of nanomaterials deserves further study. Some studies have reported that the nanomaterials used for treatment have no side effects on the survival of normal endometrial cells and the body weights of experimental animals. However, nanodrugs are usually injected into experimental animals through the tail vein, and it is not clear whether they will affect other the tissues and organs, and it is especially important to determine whether the nanomaterials will affect their long-term health and the health of their offspring. Thus, we should further strengthen the research on the safety of nanomaterials, optimize their performance, and promote their application in the treatment of endometriosis.

Although nanotechnology has had a significant effect on the treatment of endometriosis in animal models, more research and exploration are still needed for its clinical application. According to our best knowledge, no clinical trials have been conducted on the applications of nanomaterials for the treatment of endometriosis. There are a series of challenges that limit the transition of nanomedicines from bench to bedside, such as a lack of extensive research data support and significant financial support, inability to predict the prognosis of patients with endometriosis.

In summary, more research is needed to clarify that nanotechnology may have great potential in the treatment of endometriosis to obtain a favourable therapeutic effect.

## References

[B1] Acharya M. R. Venitz J. Figg W. D. Sparreboom A. (2004). Chemically modified tetracyclines as inhibitors of matrix metalloproteinases. Drug Resist Updat 7, 195–208. 10.1016/j.drup.2004.04.002 15296861

[B2] Akkaya P. Onalan G. Haberal N. Bayraktar N. Mülayim B. Zeyneloglu H. B. (2009). Doxycycline causes regression of endometriotic implants: A rat model. Hum. Reprod. 24, 1900–1908. 10.1093/humrep/dep106 19401321

[B3] Anbil S. Rizvi I. Celli J. P. Alagic N. Pogue B. W. Hasan T. (2013). Impact of treatment response metrics on photodynamic therapy planning and outcomes in a three-dimensional model of ovarian cancer. J. Biomed. Opt. 18, 098004. 10.1117/1.Jbo.18.9.098004 24802230 PMC3783041

[B4] Andaloussi S. E. Lehto T. Mäger I. Rosenthal-Aizman K. Oprea II Simonson O. E. (2011). Design of a peptide-based vector, PepFect6, for efficient delivery of siRNA in cell culture and systemically *in vivo* . Nucleic Acids Res. 39, 3972–3987. 10.1093/nar/gkq1299 21245043 PMC3089457

[B5] Arablou T. Kolahdouz-Mohammadi R. (2018). Curcumin and endometriosis: Review on potential roles and molecular mechanisms. Biomed. Pharmacother. 97, 91–97. 10.1016/j.biopha.2017.10.119 29080464

[B6] Becker C. M. Sampson D. A. Short S. M. Javaherian K. Folkman J. D'Amato R. J. (2006). Short synthetic endostatin peptides inhibit endothelial migration *in vitro* and endometriosis in a mouse model. Fertil. Steril. 85, 71–77. 10.1016/j.fertnstert.2005.07.1290 16412733

[B7] Boroumand S. Hosseini S. Pashandi Z. Faridi-Majidi R. Salehi M. (2019). Curcumin-loaded nanofibers for targeting endometriosis in the peritoneum of a mouse model. J. Mater Sci. Mater Med. 31, 8. 10.1007/s10856-019-6337-4 31838602

[B8] Celardo I. Traversa E. Ghibelli L. (2011). Cerium oxide nanoparticles: A promise for applications in therapy. J. Exp. Ther. Oncol. 9, 439–448. 21275265

[B9] Chaudhury K. Babu K. N. Singh A. K. Das S. Kumar A. Seal S. (2013). Mitigation of endometriosis using regenerative cerium oxide nanoparticles. Nanomedicine 9, 439–448. 10.1016/j.nano.2012.08.001 22960424

[B10] Choo Y. W. Kang M. Kim H. Y. Han J. Kang S. Lee J. R. (2018). M1 macrophage-derived nanovesicles potentiate the anticancer efficacy of immune checkpoint inhibitors. ACS Nano 12, 8977–8993. 10.1021/acsnano.8b02446 30133260

[B11] Clower L. Fleshman T. Geldenhuys W. J. Santanam N. (2022). Targeting oxidative stress involved in endometriosis and its pain. Biomolecules 12, 1055. 10.3390/biom12081055 36008949 PMC9405905

[B12] Dad H. A. Gu T. W. Zhu A. Q. Huang L. Q. Peng L. H. (2021). Plant exosome-like nanovesicles: Emerging therapeutics and drug delivery nanoplatforms. Mol. Ther. J. Am. Soc. Gene Ther. 29, 13–31. 10.1016/j.ymthe.2020.11.030 PMC7791080 33278566

[B13] Dass C. R. Contreras K. G. Dunstan D. E. Choong P. F. (2007). Chitosan microparticles encapsulating PEDF plasmid demonstrate efficacy in an orthotopic metastatic model of osteosarcoma. Biomaterials 28, 3026–3033. 10.1016/j.biomaterials.2007.03.016 17408737

[B14] de Almeida Borges V. R. Tavares M. R. da Silva J. H. Tajber L. Boylan F. Ribeiro A. F. (2018). Development and characterization of poly(lactic-co-glycolic) acid nanoparticles loaded with copaiba oleoresin. Pharm. Dev. Technol. 23, 343–350. 10.1080/10837450.2017.1290107 28145793

[B15] Duong T. Li X. Yang B. Schumann C. Albarqi H. A. Taratula O. (2017). Phototheranostic nanoplatform based on a single cyanine dye for image-guided combinatorial phototherapy. Nanomedicine 13, 955–963. 10.1016/j.nano.2016.11.005 27884637

[B16] Fang Z. Zhang X. Huang H. Wu J. (2022). Endometriosis. Chin. Chem. Lett. 33, 1244–1256.

[B17] Foged C. Nielsen H. M. Frokjaer S. (2007). Liposomes for phospholipase A2 triggered siRNA release: Preparation and *in vitro* test. Int. J. Pharm. 331, 160–166. 10.1016/j.ijpharm.2006.11.010 17156952

[B18] Greaves E. Cousins F. L. Murray A. Esnal-Zufiaurre A. Fassbender A. Horne A. W. (2014). A novel mouse model of endometriosis mimics human phenotype and reveals insights into the inflammatory contribution of shed endometrium. Am. J. Pathol. 184, 1930–1939. 10.1016/j.ajpath.2014.03.011 24910298 PMC4076466

[B19] Guidotti G. Brambilla L. Rossi D. (2017). Cell-penetrating peptides: From basic research to clinics. Trends Pharmacol. Sci. 38, 406–424. 10.1016/j.tips.2017.01.003 28209404

[B20] Guo S. W. Hummelshoj L. Olive D. L. Bulun S. E. D'Hooghe T. M. Evers J. L. (2009). A call for more transparency of registered clinical trials on endometriosis. Hum. Reprod. 24, 1247–1254. 10.1093/humrep/dep045 19264712 PMC4102915

[B21] Guo X. Li W. Zhou J. Hou W. Wen X. Zhang H. (2017). Specific photothermal ablation therapy of endometriosis by targeting delivery of gold nanospheres. Small 13, 1603270. 10.1002/smll.201603270 28145630

[B22] Gupta S. C. Patchva S. Aggarwal B. B. (2013). Therapeutic roles of curcumin: Lessons learned from clinical trials. Aaps J. 15, 195–218. 10.1208/s12248-012-9432-8 23143785 PMC3535097

[B23] Hansen R. R. Nasser A. Falk S. Baldvinsson S. B. Ohlsson P. H. Bahl J. M. (2012). Chronic administration of the selective P2X3, P2X2/3 receptor antagonist, A-317491, transiently attenuates cancer-induced bone pain in mice. Eur. J. Pharmacol. 688, 27–34. 10.1016/j.ejphar.2012.05.008 22634164

[B24] Henriques da Silva J. Borges V. R. Pereira Lda C. Ferrari R. de Mattos R. M. Barros E. G. (2015). The oil-resin of the tropical rainforest tree Copaifera langsdorffii reduces cell viability, changes cell morphology and induces cell death in human endometriotic stromal cultures. J. Pharm. Pharmacol. 67, 1744–1755. 10.1111/jphp.12479 26407531

[B25] Hilger I. (2013). *In vivo* applications of magnetic nanoparticle hyperthermia. Int. J. Hyperth. 29, 828–834. 10.3109/02656736.2013.832815 24219800

[B26] Horne A. W. Missmer S. A. (2022). Pathophysiology, diagnosis, and management of endometriosis. Bmj 379, e070750. 10.1136/bmj-2022-070750 36375827

[B27] Jurenka J. S. (2009). Anti-inflammatory properties of curcumin, a major constituent of curcuma longa: A review of preclinical and clinical research. Altern. Med. Rev. 14, 141–153. 19594223

[B28] Kiisholts K. Kurrikoff K. Arukuusk P. Porosk L. Peters M. Salumets A. (2021). Cell-penetrating peptide and siRNA-mediated therapeutic effects on endometriosis and cancer *in vitro* models. Pharmaceutics 13, 1. 10.3390/pharmaceutics13101618 PMC8541686 34683911

[B29] Kim B. Y. Rutka J. T. Chan W. C. (2010). Nanomedicine. N. Engl. J. Med. 363, 2434–2443. 10.1056/NEJMra0912273 21158659

[B30] Koopmans R. J. Aggeli A. (2010). Nanobiotechnology--quo vadis? Curr. Opin. Microbiol. 13, 327–334. 10.1016/j.mib.2010.01.012 20149720

[B31] Kumar C. S. Mohammad F. (2011). Magnetic nanomaterials for hyperthermia-based therapy and controlled drug delivery. Adv. Drug Deliv. Rev. 63, 789–808. 10.1016/j.addr.2011.03.008 21447363 PMC3138885

[B32] Li J. Lu W. Yang Y. Xiang R. Ling Y. Yu C. (2022). Hybrid nanomaterials for cancer immunotherapy. Advanced science (Weinheim, Baden-Wurttemberg, Germany): e2204932. 10.1002/advs.202204932 PMC9951325 36567305

[B33] Li Q. Yuan M. Jiao X. Huang Y. Li J. Li D. (2021). M1 macrophage-derived nanovesicles repolarize M2 macrophages for inhibiting the development of endometriosis. Front. Immunol. 12, 707784. 10.3389/fimmu.2021.707784 34354711 PMC8329654

[B34] Li W. N. Hsiao K. Y. Wang C. A. Chang N. Hsu P. L. Sun C. H. (2020). Extracellular vesicle-associated VEGF-C promotes lymphangiogenesis and immune cells infiltration in endometriosis. Proc. Natl. Acad. Sci. U. S. A. 117, 25859–25868. 10.1073/pnas.1920037117 33004630 PMC7568311

[B35] Liang Z. Chen Y. Zhao Y. Xu C. Zhang A. Zhang Q. (2017). miR-200c suppresses endometriosis by targeting MALAT1 *in vitro* and *in vivo* . Stem Cell Res. Ther. 8, 251. 10.1186/s13287-017-0706-z 29116025 PMC5678601

[B36] Lingasamy P. Tobi A. Haugas M. Hunt H. Paiste P. Asser T. (2019). Bi-specific tenascin-C and fibronectin targeted peptide for solid tumor delivery. Biomaterials 219, 119373. 10.1016/j.biomaterials.2019.119373 31374479

[B37] Liu J. Chuang Y. J. Hu J. Akakuru O. U. Sun S. Chen T. (2020). Navigating nMOF-mediated enzymatic reactions for catalytic tumor-specific therapy. Mater. Horizons 7, 3176–3186. 10.1039/D0MH01225D

[B38] Liu C. Luo L. Zeng L. Xing J. Xia Y. Sun S. (2018). Porous gold nanoshells on functional NH(2) -MOFs: Facile synthesis and designable platforms for cancer multiple therapy. Small 14, e1801851. 10.1002/smll.201801851 30058139

[B39] Liu C. Shin J. Son S. Choe Y. Farokhzad N. Tang Z. (2021a). Pnictogens in medicinal chemistry: Evolution from erstwhile drugs to emerging layered photonic nanomedicine. Chem. Soc. Rev. 50, 2260–2279. 10.1039/d0cs01175d 33367452

[B40] Liu C. Sun S. Feng Q. Wu G. Wu Y. Kong N. (2021b). Arsenene nanodots with selective killing effects and their low-dose combination with ß-elemene for cancer therapy. Adv. Mater 33, e2102054. 10.1002/adma.202102054 34309925

[B41] Liu C. Xing J. Akakuru O. U. Luo L. Sun S. Zou R. (2019). Nanozymes-engineered metal-organic frameworks for catalytic cascades-enhanced synergistic cancer therapy. Nano Lett. 19, 5674–5682. 10.1021/acs.nanolett.9b02253 31361142

[B42] Liu Q. Ma P. Liu L. Ma G. Ma J. Liu X. (2017). Evaluation of PLGA containing anti-CTLA4 inhibited endometriosis progression by regulating CD4+CD25+Treg cells in peritoneal fluid of mouse endometriosis model. Eur. J. Pharm. Sci. 96, 542–550. 10.1016/j.ejps.2016.10.031 27989857

[B43] Liu T. Liu M. Zheng C. Zhang D. Li M. Zhang L. (2021c). Exosomal lncRNA CHL1-AS1 derived from peritoneal macrophages promotes the progression of endometriosis via the miR-610/MDM2 Axis. Int. J. Nanomedicine 16, 5451–5464. 10.2147/ijn.S323671 34408418 PMC8367089

[B44] Makadia H. K. Siegel S. J. (2011). Poly lactic-co-glycolic acid (PLGA) as biodegradable controlled drug delivery carrier. Polym. (Basel) 3, 1377–1397. 10.3390/polym3031377 PMC3347861 22577513

[B45] Mendes B. B. Conniot J. Avital A. Yao D. Jiang X. Zhou X. (2022). Nanodelivery of nucleic acids. Nat. Rev. Methods Prim. 2, 24. 10.1038/s43586-022-00104-y PMC9038125 35480987

[B46] Misra R. Acharya S. Dilnawaz F. Sahoo S. K. (2009). Sustained antibacterial activity of doxycycline-loaded poly(D,L-lactide-co-glycolide) and poly(ε-caprolactone) nanoparticles. Nanomedicine (Lond). 4, 519–530. 10.2217/nnm.09.28 19572818

[B47] Mochizuki M. Yamazaki S. Kano K. Ikeda T. (2002). Kinetic analysis and mechanistic aspects of autoxidation of catechins. Biochim. Biophys. Acta 1569, 35–44. 10.1016/s0304-4165(01)00230-6 11853955

[B48] Moses A. S. Demessie A. A. Taratula O. Korzun T. Slayden O. D. Taratula O. (2021). Nanomedicines for endometriosis: Lessons learned from cancer research. Small 17, e2004975. 10.1002/smll.202004975 33491876 PMC7928207

[B49] Moses A. S. Taratula O. R. Lee H. Luo F. Grenz T. Korzun T. (2020). Nanoparticle-based platform for activatable fluorescence imaging and photothermal ablation of endometriosis. Small 16, e1906936. 10.1002/smll.201906936 32250034 PMC7210057

[B50] Nisolle M. Donnez J. (2019). Reprint of: Peritoneal endometriosis, ovarian endometriosis, and adenomyotic nodules of the rectovaginal septum are three different entities. Fertil. Steril. 112, e125–e136. 10.1016/j.fertnstert.2019.08.081 31623724

[B51] Ogasawara Y. Cheng J. Tatematsu T. Uchida M. Murase O. Yoshikawa S. (2020). Long-term autophagy is sustained by activation of CCTβ3 on lipid droplets. Nat. Commun. 11, 4480. 10.1038/s41467-020-18153-w 32900992 PMC7479109

[B52] Park Y. Demessie A. A. Luo A. Taratula O. R. Moses A. S. Do P. (2022). Targeted nanoparticles with high heating efficiency for the treatment of endometriosis with systemically delivered magnetic hyperthermia. Small 18, e2107808. 10.1002/smll.202107808 35434932 PMC9232988

[B53] Podgaec S. Rizzo L. V. Fernandes L. F. Baracat E. C. Abrao M. S. (2012). CD4(+) CD25(high) Foxp3(+) cells increased in the peritoneal fluid of patients with endometriosis. Am. J. Reprod. Immunol. 68, 301–308. 10.1111/j.1600-0897.2012.01173.x 22817851

[B54] Porosk L. Arukuusk P. Põhako K. Kurrikoff K. Kiisholts K. Padari K. (2019). Enhancement of siRNA transfection by the optimization of fatty acid length and histidine content in the CPP. Biomater. Sci. 7, 4363–4374. 10.1039/c9bm00688e 31411219

[B55] Psarros C. Lee R. Margaritis M. Antoniades C. (2012). Nanomedicine for the prevention, treatment and imaging of atherosclerosis. Nanomedicine 8 (1), S59–S68. 10.1016/j.nano.2012.05.006 22640906

[B56] Qiu J. J. Lin X. J. Zheng T. T. Tang X. Y. Zhang Y. Hua K. Q. (2019). The exosomal long noncoding RNA aHIF is upregulated in serum from patients with endometriosis and promotes angiogenesis in endometriosis. Reprod. Sci. 26, 1590–1602. 10.1177/1933719119831775 30808247

[B57] Ricci A. G. Olivares C. N. Bilotas M. A. Bastón J. I. Singla J. J. Meresman G. F. (2013). Natural therapies assessment for the treatment of endometriosis. Hum. Reprod. 28, 178–188. 10.1093/humrep/des369 23081870

[B58] Riehemann K. Schneider S. W. Luger T. A. Godin B. Ferrari M. Fuchs H. (2009). Nanomedicine--challenge and perspectives. Angew. Chem. Int. Ed. Engl. 48, 872–897. 10.1002/anie.200802585 19142939 PMC4175737

[B59] Russell P. Esser L. Hagemeyer C. E. Voelcker N. H. (2023). The potential impact of nanomedicine on COVID-19-induced thrombosis. Nat. Nanotechnol. 18, 11–22. 10.1038/s41565-022-01270-6 36536042

[B60] Sapadin A. N. Fleischmajer R. (2006). Tetracyclines: Nonantibiotic properties and their clinical implications. J. Am. Acad. Dermatol 54, 258–265. 10.1016/j.jaad.2005.10.004 16443056

[B61] Saunders P. T. K. Horne A. W. (2021). Endometriosis: Etiology, pathobiology, and therapeutic prospects. Cell 184, 2807–2824. 10.1016/j.cell.2021.04.041 34048704

[B62] Schneider L. Kalt M. Koch S. Sithamparanathan S. Villiger V. Mattiat J. (2023). BODIPY-based photothermal agents with excellent phototoxic indices for cancer treatment. J. Am. Chem. Soc. 145, 4534–4544. 10.1021/jacs.2c11650 36780327

[B63] Shakhbazau A. Isayenka I. Kartel N. Goncharova N. Seviaryn I. Kosmacheva S. (2010). Transfection efficiencies of PAMAM dendrimers correlate inversely with their hydrophobicity. Int. J. Pharm. 383, 228–235. 10.1016/j.ijpharm.2009.09.020 19770028

[B64] Simón-Gracia L. Kiisholts K. Petrikaitė V. Tobi A. Saare M. Lingasamy P. (2021). Homing peptide-based targeting of tenascin-C and fibronectin in endometriosis. Nanomater. (Basel) 11, 3257. 10.3390/nano11123257 PMC8708492 34947606

[B65] Singh A. K. Chakravarty B. Chaudhury K. (2015). Nanoparticle-assisted combinatorial therapy for effective treatment of endometriosis. J. Biomed. Nanotechnol. 11, 789–804. 10.1166/jbn.2015.2020 26349392

[B66] Singh B. N. Shankar S. Srivastava R. K. (2011). Green tea catechin, epigallocatechin-3-gallate (EGCG): Mechanisms, perspectives and clinical applications. Biochem. Pharmacol. 82, 1807–1821. 10.1016/j.bcp.2011.07.093 21827739 PMC4082721

[B67] Tan M. Li X. Zhang H. Zheng M. Xiong J. Cao Y. (2023). Förster resonance energy transfer nanobullet for photoacoustic imaging and amplified photothermal-photodynamic therapy of cancer. Adv. Healthc. Mater. 1, e2202943. 10.1002/adhm.202202943 36773308

[B68] Wang L. Dai C. Fang Y. You X. Wu J. (2022). A drug/carrier dual redox-responsive system based on 6-mercaptopurine dimer-loaded cysteine polymer nanoparticles for enhanced lymphoma therapy. Nano Res. 15, 4544–4551. 10.1007/s12274-021-4037-0

[B69] Wang N. Liu B. Liang L. Wu Y. Xie H. Huang J. (2014). Antiangiogenesis therapy of endometriosis using PAMAM as a gene vector in a noninvasive animal model. Biomed. Res. Int. 2014, 1–11. 10.1155/2014/546479 PMC4094709 25050361

[B70] Wang Z. Sun J. Qiu Y. Li W. Guo X. Li Q. (2015). Specific photothermal therapy to the tumors with high EphB4 receptor expression. Biomaterials 68, 32–41. 10.1016/j.biomaterials.2015.07.058 26264644

[B71] Wu D. Lu P. Mi X. Miao J. (2018). Exosomal miR-214 from endometrial stromal cells inhibits endometriosis fibrosis. Mol. Hum. Reprod. 24, 357–365. 10.1093/molehr/gay019 29660008

[B72] Wu P. Zhang B. Ocansey D. K. W. Xu W. Qian H. (2021). Extracellular vesicles: A bright star of nanomedicine. Biomaterials 269, 120467. 10.1016/j.biomaterials.2020.120467 33189359

[B73] Xu J. Xu J. Shi T. Zhang Y. Chen F. Yang C. (2023a). Probiotic-inspired nanomedicine restores intestinal homeostasis in colitis by regulating redox balance, immune responses, and the gut microbiome. Adv. Mater 35, e2207890. 10.1002/adma.202207890 36341495

[B74] Xu Q. Hu H. Mo Z. Chen T. He Q. Xu Z. (2023b). A multifunctional nanotheranostic agent based on Lenvatinib for multimodal synergistic hepatocellular carcinoma therapy with remarkably enhanced efficacy. J. colloid interface Sci. 638, 375–391. 10.1016/j.jcis.2023.01.144 36746055

[B75] Yang S. Wang H. Li D. Li M. (2019). Role of endometrial autophagy in physiological and pathophysiological processes. J. Cancer 10, 3459–3471. 10.7150/jca.31742 31293650 PMC6603423

[B76] Yin H. Kanasty R. L. Eltoukhy A. A. Vegas A. J. Dorkin J. R. Anderson D. G. (2014). Non-viral vectors for gene-based therapy. Nat. Rev. Genet. 15, 541–555. 10.1038/nrg3763 25022906

[B77] You X. Zhang J. Tong T. Dai C. Chen C. Wu J. (2023). Effects of polymer molecular weight on *in vitro* and *in vivo* performance of nanoparticle drug carriers for lymphoma therapy. Chin. Chem. Lett. 34, 107720.

[B78] Yuan M. Ding S. Meng T. Lu B. Shao S. Zhang X. (2017). Effect of A-317491 delivered by glycolipid-like polymer micelles on endometriosis pain. Int. J. Nanomedicine 12, 8171–8183. 10.2147/ijn.S146569 29184406 PMC5687452

[B79] Zhang L. Li H. H. Yuan M. Li D. Wang G. Y. (2020). Exosomal miR-22-3p derived from peritoneal macrophages enhances proliferation, migration, and invasion of ectopic endometrial stromal cells through regulation of the SIRT1/NF-κB signaling pathway. Eur. Rev. Med. Pharmacol. Sci. 24, 571–580. 10.26355/eurrev_202001_20033 32016958

[B80] Zhang R. You X. Luo M. Zhang X. Fang Y. Huang H. (2022). Poly(β-cyclodextrin)/platinum prodrug supramolecular nano system for enhanced cancer therapy: Synthesis and *in vivo* study. Carbohydr. Polym. 292, 119695. 10.1016/j.carbpol.2022.119695 35725183

[B81] Zhang Y. Chang X. Wu D. Deng M. Miao J. Jin Z. (2021). Down-regulation of exosomal miR-214-3p targeting CCN2 contributes to endometriosis fibrosis and the role of exosomes in the horizontal transfer of miR-214-3p. Reprod. Sci. 28, 715–727. 10.1007/s43032-020-00350-z 33048316

[B82] Zhao J. H. Wan X. Y. Xie X. Zhou C. Y. Wu Q. Y. (2006). Expression and clinical significance of Beclin1 and PTEN in endometrial carcinoma. Ai Zheng. 25: 1323–1336. 16764775

[B83] Zhao M. D. Cheng J. L. Yan J. J. Chen F. Y. Sheng J. Z. Sun D. L. (2016). Hyaluronic acid reagent functional chitosan-PEI conjugate with AQP2-siRNA suppressed endometriotic lesion formation. Int. J. Nanomedicine. 11: 1323–1336. 10.2147/ijn.S99692 27099493 PMC4821386

[B84] Zhao M. D. Li J. Q. Chen F. Y. Dong W. Wen L. J. Fei W. D. (2019). <p&gt;Co-Delivery of curcumin and paclitaxel by “core-shell” targeting amphiphilic copolymer to reverse resistance in the treatment of ovarian cancer</p&gt;. Int. J. Nanomedicine 14, 9453–9467. 10.2147/ijn.S224579 31819443 PMC6898996

[B85] Zhao M. D. Sun Y. M. Fu G. F. Du Y. Z. Chen F. Y. Yuan H. (2012). Gene therapy of endometriosis introduced by polymeric micelles with glycolipid-like structure. Biomaterials 33, 634–643. 10.1016/j.biomaterials.2011.09.077 21996531

[B86] Zhao M. Zhang M. Yu Q. Fei W. Li T. Zhu L. (2022). Hyaluronic acid-modified nanoplatforms as a vector for targeted delivery of autophagy-related gene to the endometriotic lesions in mice. Front. Bioeng. Biotechnol. 10, 918368. 10.3389/fbioe.2022.918368 35845410 PMC9283728

[B87] Zondervan K. T. Becker C. M. Missmer S. A. (2020). Endometriosis. N. Engl. J. Med. 382, 1244–1256. 10.1056/NEJMra1810764 32212520

[B88] Zou W. Chen L. (2008). Inhibitory B7-family molecules in the tumour microenvironment. Nat. Rev. Immunol. 8, 467–477. 10.1038/nri2326 18500231

